# Cost-Effective Screening for Breast Cancer Worldwide: Current State and Future Directions

**DOI:** 10.4137/bcbcr.s774

**Published:** 2008-07-02

**Authors:** A. Sarvazyan, V. Egorov, J.S. Son, C.S. Kaufman

**Affiliations:** 1 Artann Laboratories, 1459 Lower Ferry Rd., Trenton, NJ 08618, U.S.A; 2 Medical Tactile, Inc., 5757 Century Blvd., Suite 600, Los Angeles, CA 90045, U.S.A; 3 Department of Surgery, University of Washington, 2940 Squalicum Pkwy, Bellingham, WA 98225, U.S.A

**Keywords:** breast cancer, screening, cost-effectiveness, elastography

## Abstract

Affordability of healthcare is highly limited by its skyrocketing cost. Access to screening and diagnostic medical equipment and medicine in developing countries is inadequate for the majority of the population. There is a tremendous worldwide need to detect breast cancer at its earliest stage. These needs must be balanced by the ability of countries to provide breast cancer screening technology to their populations. We reviewed the diagnostic accuracy, procedure cost and cost-effectiveness of currently available technique for breast screening and diagnosis including clinical breast examination, mammography, ultrasound, magnetic resonance imaging, biopsy and a new modality for cancer diagnostics termed elasticity imaging that has emerged in the last decade. Clinical results demonstrate that elasticity imaging even in its simplest and least sophisticated versions, like tactile imaging, has significant diagnostic potential comparable and exceeding that of conventional imaging techniques. In view of many countries with limited resources, effective yet less expensive modes of screening must be considered worldwide. The tactile imaging is one method that has the potential to provide cost-effective breast cancer screening and diagnostics.

## Introduction

Affordability of healthcare is highly limited by its skyrocketing cost. Access to screening and diagnostic medical equipment and medicine in developing countries is inadequate for the majority of the population. More than 70% of all cancer deaths occur in low and middle income countries, where resources available for diagnosis, prevention, and treatment of cancer are limited or nonexistent[1, 2]. One of reasons for rapid escalation of the healthcare costs is an application of new advanced techniques for diagnostics, treatment and prevention which often is not cost-effective. For example, Medicare’s reimbursement system is establishing the minimum reimbursement for mammography services as 81.86 USD for film and 131.50 USD for digital bilateral screening mammography[3] which corresponds to 60.6% increase in the procedure cost. Despite such an increase of costs, the improvement of diagnostic accuracy of digital relative to film mammography is not significant. The overall diagnostic accuracy in a large-scaled clinical study was found of 0.78 ± 0.02 for digital mammography and of 0.74 ± 0.02 for film mammography (difference 0.03; 95% confidence interval, −0.02 to 0.08; P = 0.18)[4]. Digital mammography, compared with film, would cost more than 300,000 USD per quality-adjusted life-year gained, which is not cost effective[5].

These and other similar data indicate that there is an urgent need in cost effective screening and diagnostic methods for breast cancer, making it affordable all around the world[6].

## Current Screening and Diagnostic Methods

Current methods of breast screening and diagnosis include Breast Self-Examination (BSE), Clinical Breast Examination (CBE), Mammography, Ultrasound, Magnetic Resonance Imaging (MRI), and biopsy. Other breast screening methods which are currently in an exploratory stage include: tomosynthesis, supersonic shear wave imaging, electrical impedance tomography, optical tomography, and several second line breast pathology diagnostic techniques such as positron emission tomography and scintimammography.

### BSE

The studies of the effectiveness of BSE as a detection modality has shown mixed results, but recent data reviews have focused on the lack of direct benefit in randomized clinical trials[7–9]. The studies found no reduction in the breast cancer mortality but higher rate of benign biopsy, in women who regularly perform BSE compared to women who do not regularly perform BSE [8]. Although the American Cancer Society no longer recommends that all women perform monthly BSE, women are recommended to be informed about the potential benefits (self-awareness) and limitations (false-positive rate) associated with BSE. Women who detect their own breast cancer usually find it outside of a structured breast self-exam while bathing or getting dressed. A woman who wishes to perform periodic BSE should receive instruction from her health care provider and/or have her technique reviewed periodically[10].

### CBE

The premise underlying CBE is utilizing a trained clinician to visually inspect and palpate the breast in order to detect abnormalities to find palpable breast cancers at an earlier stage[11]. American Cancer Society guidelines recommend an annual CBE for age 40 and older for early detection of breast cancer in asymptomatic women[10]. The CBE may identify some cancers missed by mammography[12, 13] and provide an important screening tool among women for whom mammography is not recommended or who do not receive recommended high-quality screening mammography. At the same time, CBE performance, reports and documentation are inconsistent and not standardized. Health care providers report a lack of confidence in their CBE skills and would welcome training and practical recommendations for optimizing performance and reporting[14].

Data from six studies examined by Barton et al. resulted in an overall estimate of 54.1% for CBE sensitivity and 94.0% for CBE specificity[15]. Over 20 years ago, Haagensen [16] estimated that 65% of 2,198 breast cancer cases, identified before the use of screening mammography, presented as a breast masses detected by BSE or CBE. These findings are comparable to the published values for CBE sensitivity (58.8%) and specificity (93.4%) observed in the U.S. national screening program for 752,081 CBE reports[17]. The CBE cost effectiveness in cancer screening is 3.5 fold better than that of mammography[18]. The CBE detects only 34% fewer breast cancers than mammography, as it was demonstrated for population of 1 million women and the cost-effectiveness of biennial CBE is evaluated as 522 USD per life-year saved in India [18]. From this point, CBE may be a suitable option for countries in economic transition, where incidence rates are on the increase but limited resources do not permit screening by mammography.

In Japan, for women aged 40–49 years, having the highest incidence rate of breast cancer, the cost-effectiveness of annual CBE per life-year was evaluated as 31,900 USD[69].

### Mammography

Mammography provides X-ray images of the breasts with at least two sets of images, the mediolateral oblique and cranial-caudal views. A recent large-scale clinical study (42,760 patients in U.S.A. and Canada) on the diagnostic performance of mammography for breast-cancer screening demonstrated a sensitivity of 70.0%, specificity of 92.0%, and diagnostic accuracy interpreted as AUC of 78.0%[4]. The European randomized mammography screening trial (23,929 patients in Norway) revealed a sensitivity of 77.4% and specificity of 96.5% at full-field digital mammography. The median size of screening-detected invasive cancers was about 13.5 mm[19]. In the United States, despite the recommendation for an annual mammogram, in 2005 only 47.8% of women aged 40–49 years had a mammogram within the past year. Among the women without health insurance coverage this value decreases to 24.1% [10]. The cost-effectiveness screening film mammography are estimated as 902–1,946 USD per year of life saved in India, 2,450–14,790 USD per year of life saved in Europe, and 28,600–47,900 USD per year of life saved in U.S.A.[6]. Among the limitations of mammography are increased breast density, technical factors, e.g. areas adjacent to the chest wall may not be imaged[20], lack of insurance coverage, disagreements among primary care physicians on frequency of mammographic screening, variation in interpretation skills of radiologists.

The mean glandular radiation dose from 2-view mammography is approximately 4 to 5 mGy and the dosage varies among facilities and increases with breast density. The average cumulative exposure from screening during the decade will be around 60 mGy[70]. There is a strong linear trend of increasing risk of radiation-induced breast cancer with increasing radiation dose (P = 0.0001) [71]. A statistically significant increase in the incidence of breast cancer following radiation treatment of various benign breast diseases was observed[72]. Several recent studies suggesting that carriers of pathogenic alleles in DNA repair and damage recognition genes may have an increased risk of breast cancer following exposure to ionizing radiation, even at low doses[73]. Based on review of 117 studies related to screening mammography the authors concluded that “the risk for death due to breast cancer from the radiation exposure involved in mammography screening is small and is outweighed by a reduction in breast cancer mortality rates from early detection.”[74].

### Ultrasound

Ultrasonography as an imaging tool uses sound waves that pass through breast tissue and are reflected back characterizing tissue structure. Ultra-sonography is typically used as a complementary method for the assessment of mammographically or clinically detected breast masses and for supplemental information on dense tissue[11]. However, there is limited data supporting the use of ultrasound in breast cancer screening as an adjunct to mammography[21]. The conventional ultrasound is more often used to evaluate an area of concern on mammogram. The majority of cystic masses are benign while solid masses need further evaluation[22]. Ultrasound is often confused as a screening tool by both patients and healthcare providers. However, ultrasonic screening the entire breast is not only labor-intensive, but operator-dependent; therefore, ultrasound is a difficult tool to use if there is not an identifiable area of concern. Ongoing studies are trying to determine whether there is a population of women who would benefit from an ultrasonic screening; however, at this time, it is not the standard of care and whole-breast ultrasonography for screening has not been established as useful[23]. The cost associated with unilateral or bilateral ultrasound diagnostic procedure is 70.11 USD according to 2005 U.S. average Medicare reimbursements[5].

### MRI

MRI utilizes magnetic fields to produce detailed cross-sectional images of the breast tissue. Image contrast between tissues in the breast (fat, glandular tissue, lesions, etc.) depends on the mobility and magnetic environment of the hydrogen atoms in water and fat that contribute to the measured signal that determines the brightness of tissues in the image. Many indications for clinical breast MRI are recognized, including resolving findings on mammography and staging of breast cancer[22]. Overall, the results of 6 nonrandomized prospective studies in the Netherlands[24], the United Kingdom[25], Canada[26], Germany[27], the United States[28], and Italy[29] of MRI efficacy in breast cancer screening for high risk women populations demonstrate an averaged sensitivity of 87.5% and specificity of 92.8%. Only limited data are available on the cost effectiveness of breast MRI screening being combined with mammography. The cost per quality-adjusted life year saved for annual MRI plus film mammography, compared with annual film mammography alone, varied by age and other factors to be found in the range of 27,544–130,420 USD. The reimbursement for bilateral MRI diagnostic procedures was 1,037 USD according to 2005 U.S. average Medicare reimbursements, which is about eight times higher than the screening mammography[5] and out of pocket charges by private clinics are as much as 5 times higher.

### Ultrasound and MR elasticity imaging

In the last decade a new modality for cancer diagnostics termed Elasticity Imaging (EI) has emerged. EI allows visualization and semi quantitative assessment of mechanical properties of soft tissue. Mechanical properties of tissues, i.e. is elastic modulus and viscosity, are highly sensitive to tissue structural changes accompanying various physiological and pathological processes. A change in Young’s modulus of tissue during the development of a tumor could reach thousands of percent [30–32]. EI is based on generating a stress in the tissue using various static or dynamic means and measuring resulting strain by ultrasound or MRI [33–39]. The current increasing flow of publications from many countries all over the world on Elastography covers practically all key human organs[40–46].

### Tactile imaging

Tactile Imaging (TI), an alternative version of Elasticity Imaging, yields a tissue elasticity map, similarly to other elastographic techniques. At the same time, TI, which is also called “stress imaging” or “mechanical imaging” [56–61], most closely mimics manual palpation, since the TI probe with a pressure sensor array mounted on its face acts similar to human fingers during clinical examination, slightly compressing soft tissue by the probe.

There are limited clinical data on diagnostic/screening potential of breast TI. In one clinical study that included 110 patients with a complaint of a breast mass, TI demonstrated detection of 94% of the breast mass, while physical examination identified only 86%[57]. The positive predictive value for breast cancer using TI was 94% and 78% for physical examination. Clinical results of another study for 187 cases, collected at 4 different clinical sites, have demonstrated that TI produces a reliable image formation of breast tissue abnormalities with increased hardness and calculation of lesion features[60]. Malignant breast lesions (histologically confirmed) demonstrated increased hardness and strain hardening as well as decreased mobility and relative boundary length in comparison with benign lesions. Statistical analysis of the TI differentiation capability for 154 benign and 33 malignant lesions revealed an average sensitivity of 89.4% and specificity of 88.9% with a standard deviation of ±7.8%. The area under the receiver operating characteristic curve characterizing benign and malignant lesion discrimination was 87.8% with the confidence interval range from 82.1% to 92.1%, with a significance level P = 0.0001.

In Table 1 we summarized recent clinical results on benign/malignant breast lesion differentiation by various elasticity imaging modalities: USE—Ultrasound Elastography, MRE—Magnetic Resonance Elastography and TI. These data show that elasticity imaging even in its simplest and least sophisticated versions, like TI, has significant diagnostic potential comparable and exceeding that of conventional imaging techniques such as mammography, MRI and ultrasound.

### Biopsy

Although the most of women who undergo screening each year do not have breast cancer, about 5%–10% of women have their mammogram interpreted as abnormal or inconclusive until further tests are done. In most instances, additional tests (imaging studies and/or biopsy) lead to a final interpretation of normal breast tissue or benign [10]. In the United States alone, more than 1 million breast biopsies are performed annually and approximately 80% of these findings are benign[62, 63]. In general, the biopsy diagnostic cancer sensitivity varies from 91% to 100% (in average 96.6%) for 8 clinical trials, and depends on biopsy type (needle, core, or surgical) and used image–guided technique (X-rays, ultrasound, MRI)[64]. The evaluations of cost effectiveness of biopsy are extremely diverse depending on biopsy type, used technique, and accepted model; it is varying from 2,250 USD to 77,500 USD per life year saved [65, 66]. The cost associated with biopsy diagnostic procedure is in average about 1,000 USD, changing from 456 USD for fine needle aspiration biopsy to 2,061 USD for open biopsy, according to 2005 US average Medicare reimbursements[5].

## Cost-Effectiveness of Breast Cancer Screening and Diagnostic Methods

About 80% of the 1.6B women live in developing countries and 70% of breast cancer related deaths occur in these regions [2]. However, less than 10% of mammograms are conducted in these developing regions (Fig. 1) [67].

Diagnostic efficacy is certainly an important measure, but affordability is another critical factor which needs to be considered. Based on data from [62] we see that the adoption of mammography is strongly correlated with physician income and consequently much more utilized in developing counties. As Figure 2 shows, the number of mammography equipment in comparison to number of physicians that perform CBE is close to 20% in U.S.A. but less than 0.25% in India.

Table 2 presents a summary of breast cancer screening/diagnostic efficiency for various techniques, procedure cost and cost-effectiveness numbers. The cost-effectiveness data listed in the table are mostly taken from published sources. We included the range for cost-effectiveness for analyzed modalities in the Table 2 because the specific data depend on accepted population-based model simulating histories of women, which, as a rule, include breast cancer natural history, breast cancer detection capability of the modality, breast cancer treatment, and competing-cause mortality. Different authors often use different models for the cost-effectiveness evaluation.

The ‘gold standard’ in cancer diagnostics, biopsy, demonstrates the highest diagnostic accuracy close to 100%. It costs in average over 2,000 USD for one analyzed breast lesion/location. The biopsy cost-effectiveness varies from 2,250 USD for developing countries to 77,500 USD for developed countries. The cost-effectiveness of biennial film mammography screening is evaluated as 1,846 USD per life-year saved in India[18]. In the United States, the selective use of digital mammography screening for women aged 40 years or older had costs per quality adjusted life-year (QALY) gained ranging from 26,500 USD for age-targeted digital mammography to 84,500 USD for age- and density-targeted digital mammography. All-digital mammography screening was also more costly and no more effective than age-targeted digital mammography. The cost per QALY gained for all-digital mammography relative to all-film mammography screening was 331,000 USD (Confidence interval, 268,000 USD to 403,000 USD)[4].

Using cost-effectiveness of biennial CBE, 522 USD per life-year saved in India[18], one can estimate the impact of TI using CBE data. Taking 92% TI cancer sensitivity, which is 62% higher than CBE, may result in proportional improvement in TI cost-effectiveness. Further, the TI examination may be performed by a nurse or qualified technician, rather than a physician, which may additionally improve the cost effectiveness by a factor of two. As a result, we may expect TI cost effectiveness for developing countries to be equaled to 162 USD (in prices of 2001), which is over ten times more cost-effective than film mammography. A rough estimate shows that the TI procedure could cost about 5 USD in developing countries and 50 USD in the United States. In addition, clinical results indicate that TI screening may substantially decrease the benign biopsy rate[60].

## Conclusions

There is a tremendous worldwide need to detect breast cancer at its earliest stage. These needs must be balanced by the ability of countries to provide breast cancer screening technology to their populations. We reviewed the current available screening and diagnostic techniques with their relative cost-effectiveness ratios. In view of many countries with limited resources, effective yet less expensive modes of screening must be considered worldwide. Tactile imaging is one method that has the potential to provide cost-effective breast cancer screening and diagnostics.

## Figures and Tables

**Figure 1.**
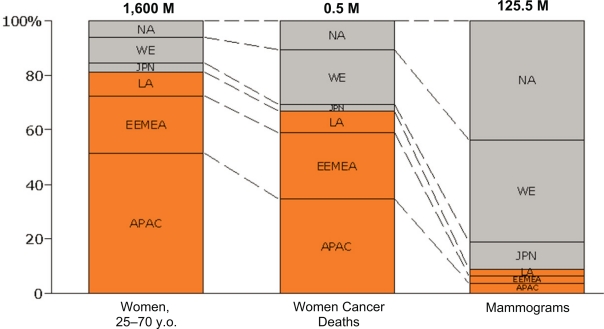
Population of women (first column), breast cancer deaths (second column) and mammography processes (third column) in developing countries (red boxes) in comparison with developed countries (grey boxes). The data are for 2005 [62]. **Abbreviations:** NA: North America; WE: Western Europe; JPN: Japan; LA: Latin America; EEMEA: Eastern Europe, Middle East, Africa; APAC: Asian Pacific, Australia, China.

**Figure 2.**
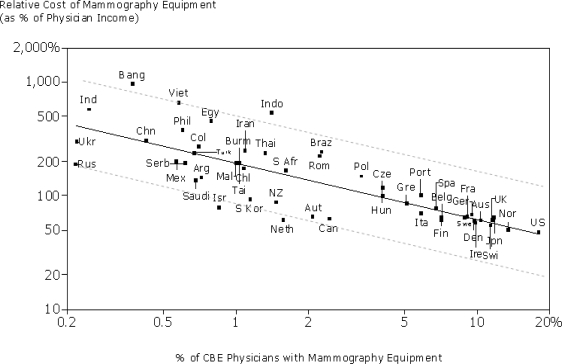
Relative cost of mammography equipment vs. physician adoption.

**Table 1.** Recent clinical data on benign-malignant breast lesion differentiation by elasticity imaging.

**No.**	**Method**	**Number of analyzed lesions**	**Sensitivity**	**Specificity**	**Citation**
1	USE*	52 malignant	**86.5%**	**89.8%**	Itoh A, et al. 2006 [47]
		59 benign			
2	USE	135 total	**100.0%**	**95.0%**	Zhang XF, et al. 2006 [48]
3	USE	49 malignant	**91.8%**	**91.5%**	Thomas A, et al. 2006 [49]
		59 benign			
4	MRE*	38 malignant	**95.0%**	**80.0%**	Sinkus R, et al. 2006 [50]
		30 benign			
5	USE	88 total	**96.0%**	**61.0%**	Renger DM, et al. 2006 [51]
6	USE	43 malignant	**100.0%**	**96.0%**	Barr RG, 2007 [52]
		150 benign			
7	USE	115 total	**90.0%**	**–**	Garra BS, et al. 2006 [53]
8	USE	50 malignant	**99.3%**	**25.7%**	Burnside ES, et al. 2007 [54]
		48 benign			
9	USE	237 malignant	**97.5%**	**48.0%**	Svensson WE, et al. 2007 [55]
		584 benign			
10	TI*	34 malignant	**94.4%**	**–**	Kaufman CS, et al. 2006 [57]
		76 benign			
11	TI	33 malignant	**89.4%**	**88.9%**	Egorov V, et al. 2008 [60]
		154 benign			
12	SSI*	4 malignant	**100.0%**	**100.0%**	Tanter M, et al. 2008 [75]
		11 benign			

USE*—Ultrasound Elastography, MRE*—Magnetic Resonance Elastography, TI*—Tactile Imaging, SSI*—Supersonic Shear Imaging.

**Table 2.** Comparative data for breast cancer detection effectiveness and cost effectiveness.

**Screening/Diagnostic technique**	**Sensitivity/Specificity, %**	**Procedure cost of ilateral exam, USD**	**Cost-effectiveness, USD per life year gained**
CBE	56.5/93.7	–	522, India [7]
			31,900, Japan [69]
Mammography	73.7/94.3	112*	1,846, India [7]
			26,500–331,000 [5]
Ultrasound	Limited, see text	70*	–
MRI	87.7/92.8	1,037*	55,420–130,695 [68]
Biopsy	96.6/100.0	2,061**	2,250–77,500 [65, 66]
Elasticity Imaging	95.1^#^/73.4	–	–
Tactile Imaging	91.9^##^/88.9	5–50***	162***

* the U.S. average Medicare reimbursements in 2005;

** in average for one biopsy;

*** projections based on a physician’s assistant performing the exam;

# averaged for 9 clinical studies;

## averaged for 2 clinical studies.
